# Nutritional Status and Humoral Immune Response to *Plasmodium falciparum* in Children Aged 6–59 Months

**DOI:** 10.1155/2020/1843780

**Published:** 2020-05-20

**Authors:** Arnaud Tepa, Idrissa Abame, Viviane Makamta, Balotin Fongang, Josiane Donkeu, Lawrence Ayong, Constant Anatole Pieme

**Affiliations:** Faculty of Medicine and Biomedical Sciences of the University of Yaoundé 1, Yaounde, Cameroon

## Abstract

Malaria is a leading cause of morbidity and mortality in Africa. Children are mostly exposed to this disease; numerous studies have investigated the relationship between child malnutrition and either malaria morbidity or infection. Few studies demonstrated the interaction between child malnutrition and specific anti-*Plasmodium falciparum* immune responses. The purpose of this study was to investigate the impact of nutritional status and iron on total anti-*Plasmodium falciparum* IgG levels in children living in the Gado-Badzéré refugee camp. We carried out a cross-sectional study during August–November 2017 in the Gado-Badzéré refugee camp in the East region of Cameroon. Children aged from 6 to 59 months with fever were recruited from the medical center. The data were recorded using a standardized data collection sheet and were analyzed using SPSS and WHO Anthro software. The total anti-Pf 3D7 total IgG level was determined using an ELISA technique while a colorimetric method was used to measure the total iron level. A total of 83 patients aged 6–59 months were enrolled in this study. The prevalence of malaria and malnutrition was 47% and 31%, respectively. Acute malnutrition was statistically less recurrent in noninfected children compared with that in the infected children. The infection tended to have significant influence on the level of anti-*Plasmodium falciparum* antibodies in children. In addition, nutritional status and serum iron levels had no significant influence on children's anti-Pf IgG *T* levels. Malaria and malnutrition remain real public health problems in the Gado-badzéré refugee camp. Knowledge of the nutritional profile of the population would be of great benefit in setting up an appropriate health program. We therefore suggest that more standardized studies be conducted to highlight the effect of nutrition and micronutrients on immunological status.

## 1. Introduction

Malaria is a parasitic disease transmitted to humans by the bite of an infected female *Anopheles* mosquito. Out of the five species of *Plasmodium*, *Plasmodium falciparum* is the most prevalent malaria parasite in sub-Saharan Africa, accounting for 99.7% of estimated malaria cases in 2018 [1]. Estimation from the World Health Organization (WHO) indicates there were over 228 million new malaria cases and 405 000 deaths across all age groups in 2018 [1]. It is a leading cause of morbidity and mortality in sub-Saharan Africa; 93% of malaria cases worldwide occur in sub-Saharan Africa alone, followed by the South-East Asia region (3.4%) and the WHO Eastern Mediterranean Region (2.1%) [1]. In Cameroon, the prevalence of malaria was estimated at 27% at 2017 [2]. Malaria is an important cause of anemia in endemic regions through the destruction of parasitized red blood cells, increased clearance of infected, and uninfected red blood cells by the spleen, cytokine-induced dyserythropoiesis, and probably decreased absorption of dietary iron [3]. It had been highlighted that iron levels influence the appearance and the evolution of malarial infection by interacting with the immune system and the parasite [4–7]. Numerous studies have investigated the relationship between child malnutrition and either malaria morbidity or intensity of infection (4,5). In contrast, only a few studies have explored the interaction between child malnutrition and specific anti-*Plasmodium falciparum* (anti-Pf) immune responses. Moreover, results of those studies are conflicting [8]. Taking into account the results of the survey conducted by Chiabi et al. in 2014, the Gado-Badzéré refugee camp is one of the areas with high malaria prevalence (39%) where malnutrition is also high (15%) [9]. In order to know if there is a relationship between the nutritional status and IgG production in this area, we carried out a study to investigate the impact of nutritional status on total anti-*Plasmodium falciparum* IgG levels in children living in the Gado-Badzéré refugee camp.

## 2. Methods

### 2.1. Study Design and Population

We carried out a cross-sectional study between November and December 2017 at the medical Centre of the Gado-Badzéré refugee camp located in the eastern region of Cameroon, Department of Lom-et-Djerem, 75 kms from the border with Central African Republic (CAR). We included in the study all children aged from 6 to 59 months with fever admitted in the Gado-badzéré medical center. No noninclusion or exclusion criteria were applied to this study. Sampling was done using the nonprobability technique of convenience, taking into account only those children who met the criteria listed above. The sample size was estimated using the following equation (*n* = *D* ((Z*α* + Z*β*)^2^ ∗ (P1 (1−P1) + P2 (1−P2))/(P2–P1)^2^)) as described by Magnani (2001) [10], where P_1_ represents the estimated prevalence of malaria in the Gado-badzéré refugees camp and P_2_ represents the estimated prevalence of severe acute malnutrition in this camp.

Nutritional assessment was done through the anthropometric data collected during consultation. Weight measurements were taken using baby scales (SECA, Hamburg, Germany), for children weighing less than 16 and an electronic scale (Téfal, Paris, France). Recumbent length measurements were taken for children under 2 years of age, while standing height was measured beyond that age. Body mass index (BMI) was calculated using the program ANTHRO and ANTHRO PLUS(11). BMI for age z scores below minus two was defined as undernutrition. Growth velocity was measured in cm/year, defined as the difference between the final and initial height in the period of 12 months. The classification of each individual as having adequate or inadequate growth was made according to the WHO standards for each age [12].

### 2.2. Collection and Processing of Blood Samples

To assess the prevalence of malaria, the malaria rapid diagnostic test (RDT) was collected, and the positive result was confirmed by blood for thick/thin smears.

Blood smears were prepared on the same slide bearing a patient's identification code as described by Maketa et al. (2015) [13]. Iron concentration was measured in the laboratory using the Biolabo® device kit (SFBC, French) with venous blood collected in the field and stored in vacutainer tubes containing no anticoagulant.

### 2.3. Cultivation of Parasites

*Plasmodium falciparum* strain 3D7 was cultured, as previously described [14]. More precisely, the culture was made using a base medium: RPMI 1640 (1x) of 500 ml containing glutamax (0.5%), glucose (20%), and sodium bicarbonate (20 mM). The preparation of the complete medium consisted of 457.3 ml RPMI added to a volume of 42.7 ml of complement (12.5 ml HEPES 23 mM + 5 ml hypoxantine1x + 200 *μ*l gentamicin 50 *μ*g/ml + 25 ml albumax 10%). An equal volume of complete medium was added to the washed group O blood (RBC 50% RPMI). In parallel, the parasites thawed in the water bath were then transferred into a 15 ml falcon tube. Five to seven drops of 12% Nacl were added to this tube and then left to rest for five minutes and centrifuged at 2500 rpm for four minutes. Then, 4.5 ml of Nacl 0.9% glucose 20% was added, mixed, and centrifuged for four minutes at 2500 rpm. After this preparation of the parasite, 5 ml of complete medium was added, mixed, and transferred to a Petri dish. Finally, 300 *μ*L of RBC 50% RPMI was added and mixed. Incubation was done in a jar. The cultures were monitored until a parasitaemia of 8–10% was obtained.

### 2.4. Extraction of Total Proteins from *Plasmodium falciparum* Strain 3D7

This step consisted initially in centrifuging the culture at 3000 rpm for five minutes at 4°C and rejecting the supernatant. The tube was washed with 10 ml of PBS 1X. Then, we suspended again the product in a mixture of PBS 1X and a 0.1% saponin solution in the PBS with equal volume. This suspension was incubated for ten minutes at 4°C. After correctly homogenizing and vortexing, the mixture was centrifuged at 4500 rpm at 4°C for ten minutes. The supernatant obtained was discarded, and the pellet was washed three times with the refrigerated PBS. A preparation of parasite lysis buffer and protease inhibitor (10X) was added to the tube, five times the volume of the pellet. This mixture was carefully vortexed for three minutes and then centrifuged for 15 minutes at 40°C at 10,000 rpm. The supernatant containing the total proteins of *P. falciparum* 3D7 was finally collected and quantified.

### 2.5. ELISA with Total Protein Extracts of *Plasmodium falciparum* Strain 3D7

Each well of the plate has been fixed with 100 µl of total protein extract prediluted in CBS solution (carbonate coating buffer solution: 0.1 M; pH 8.5). The plate was incubated for 16 hours at 4°C after being covered with parafilm paper. The plate was then washed three times with PBS 1X and wrung out on paper. The plate was fortified with 250 *μ*L of BSA/PBS 1X (pH 7.2) and incubated for one hour at room temperature and then washed three times with 250 *μ*L/well of PBS Tween 0.05% (500 *μ*L of Tween 20 in 1L of PBS 1X). Once dry, the plate was incubated with 100 *μ*L/well of diluted serum (1/250) using a solution of PBS 1X/Tween 20 BSA (0.1%) (250 ml PBS1 X + 0.25 g BSA + 125 *μ*L of Tween). After one hour of incubation at room temperature, the plate was washed five times with 250 *μ*L/well of PBS Tween 0.05%. After draining the plate, we incubated it with 100 *μ*L/well of 1/1000 prediluted peroxidase-conjugated IgG solution in PBS/TB (0.1%) (1 *μ*L conjugated IgG + 10 *μ*L PBS/TB). After one hour of incubation at room temperature, the plate was washed five times with PBS/T. The next step was to incubate the plate for ten minutes in the dark with 100 *μ*L/well of TMB substrate solution (2.2′, 5.5′-tetramethylbenzidine) for revelation. The reaction was stopped with 50 *μ*l of 2M (H_2_SO_4_) 1/10 sulphuric acid (9 *μ*l distilled water mixed with 1 ml of H_2_SO_4_). Finally, the plate was read to the ELISA plate reader (Multiskan Microplaque Photometer) to measure the absorbance at 450 nm.

### 2.6. Statistical Analysis

The data collected were captured, stored, and analyzed using Microsoft EXCEL 2010, SPSS version 20.0, and WHO Anthro software. We used descriptive statistics to summarize the distribution of quantitative and qualitative variables. The generalized linear model was used to highlight the effect of nutritional status and iron on total anti-Pf IgG levels. The results were presented in graphs and tables. The observations were considered significant for a *P* value < 0.05.

### 2.7. Ethical Considerations

Our study benefited from the ethical clearance N° 0685/CRERSHC/2017 issued by the Regional Ethics Committee for Research in the Humanities and from a UNHCR authorization N° CMR/HCR/MGA/2017/04 used for our integration into the Gado-Badzéré refugee camp in order to guarantee the transparency of our research and to avoid scientific misconduct. We have ensured that the confidentiality and anonymity of the information collected are maintained at the time of data collection and analysis.

## 3. Results

### 3.1. General Characteristics of the Study Population

#### 3.1.1. General Information

**Figure 1:**
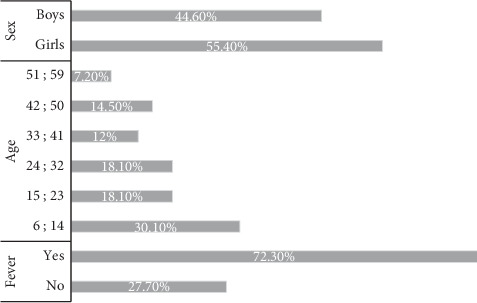
Distribution of the population by gender, age, and temperature.

A total of 83 patients were enrolled in this study. Girls represented 55% of our population compared with 45% of boys. Children aged from 51 to 59 months were the most representative age group in our study population. We observe that more than 70% of children had fever (high temperatures above 37.5°C) (Figure 1).

#### 3.1.2. Assessment of the Nutritional Status of the Study Population

**Figure 2:**
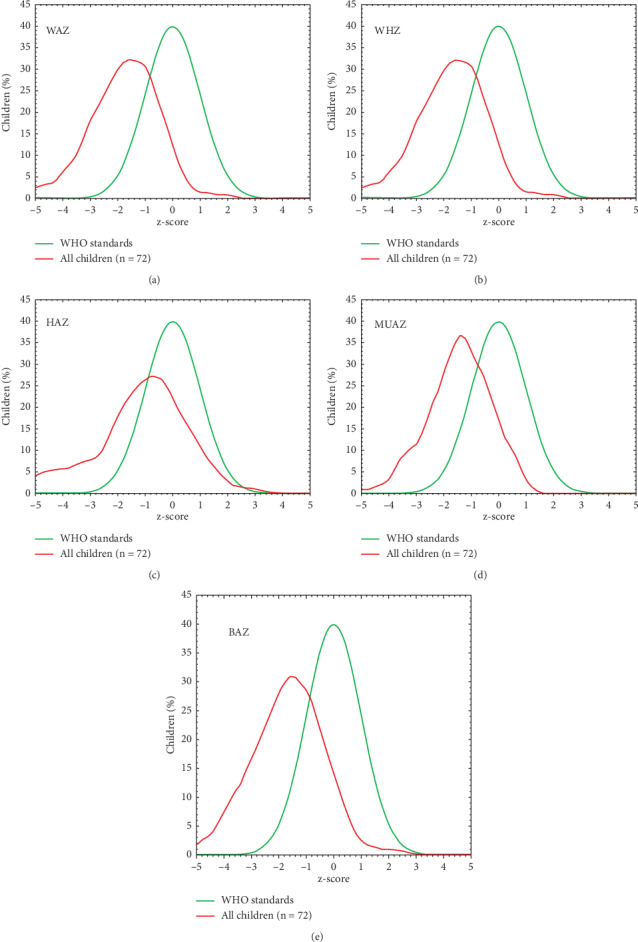
Distribution of z scores in the survey population compared to the reference population.

**Figure 3:**
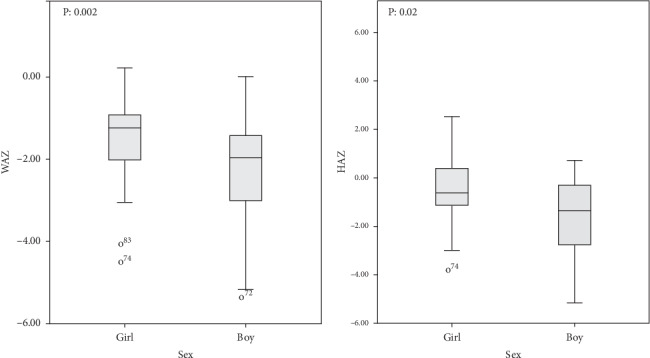
WAZ score and HAZ score as a function of gender among children in the Gado-badzéré refugee camp.

A high proportion of children had *z* scores < −2, respectively, 36.1%, 20.5%, 36.1%, 34.9%, and 28.9% for WHZ, HAZ, WAZ, BAZ, and MUACZ.  Figure 2 shows the distribution of these different *z* scores in the population based on WHO standards. The reference population appears in green and the survey population in red. Refugee children had a nutritional status below WHO standards. Acute (WAZ) and chronic (HAZ) malnutrition was statistically more recurrent among boys than girls (Figure 3). No significant differences were observed by age.

#### 3.1.3. Profile of *Plasmodium falciparum* Infection

**Figure 4:**
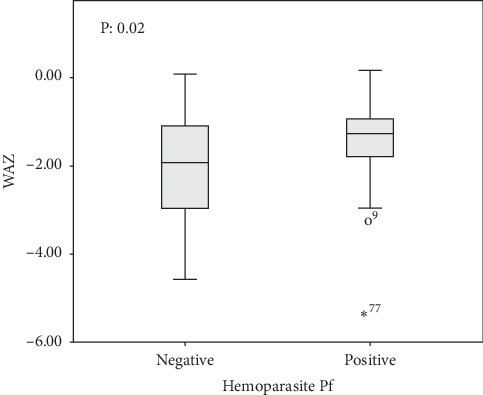
WAZ score among infected and uninfected children in the Gado-badzéré refugee camp.

The prevalence of malaria was 47% in our study population. Age and some anthropometric parameters (WHZ, HAZ, HAZ, BAZ, and MUACZ) had no significant impact on the occurrence of the disease (*P* > 0.05). On the other hand, we observed that acute malnutrition was less recurrent in noninfected children compared with that in the infected children (Figure 4).

#### 3.1.4. Total Anti-*Plasmodium falciparum* IgG Levels

For the immunoglobulin assay, a total of 44 samples were analyzed by the ELISA method to determine the total anti-*Plasmodium falciparum* IgG. The average of anti-*Plasmodium falciparum* IgG tended to be higher in children with WHZ and BAZ > −2, respectively, compared with those in whom these anthropometric parameters were <−2 (Table 1).  Table 2 represents the risk assessment for children of known nutritional or infectious status to have IgG below the estimated threshold value. Children with WHZ, HAZ, WAZ, and BAZ scores > −2, respectively, were more likely to have anti- *Plasmodium falciparum* IgG below the threshold value compared with those with these parameters < −2. However, children with a MUACZ score > −2 and those with serum iron levels >0.5 were less likely to have anti-*Plasmodium falciparum* IgG below the threshold value compared with those with a MUACZ score of < −2 and those with serum iron levels <0.5, respectively. However, these risks were not statistically significant (*P* > 0.05). *Plasmodium falciparum* infection tended to decrease the risk for patients to have an IgG level of anti-*Plasmodium falciparum* below the threshold value (RR 0.125; 95% CI 0.014–1.151; *P* = 0.06).

## 4. Discussion

A total of 83 children were recruited in our study. The main limitation of this study was the size of the sample, which was not significant. Moreover, we acknowledge that cross-sectionalnature is not very appropriate for drawing our conclusions. A longitudinal design that assesses trends in IgG changes with infection and nutritional status would be more appropriate. In addition, the inclusion of supplemented children in the study is a possible confounding factor. Of the 83 children recruited in this study, the proportion of girls was the same as that of boys. This finding is similar to that of Chiabi et al. (2016), who, in 2014, found equal number between boys and girls of the 536 children consulted in various conditions at the Gado-Badzéré refugee camp [9, 10]. The prevalence of malaria was 47% in our study population; this was slightly higher than that obtained by Chiabi et al. (2016) (39%) at a different epidemiological period (June) [9]. Indeed, our study was carried out during the high rainy season (November-December) which marks a period of high malaria prevalence compared with that in June which happens to be the end of another period of high prevalence (April-May). The age of the children, as well as anthropometric parameters such as WHZ, HAZ, BAZ, and MUACZ, had no significant impact on the occurrence of the disease. This result corroborates those of O'brien et al. (2018) who also found no association between several anthropometric parameters (HAZ, WAZ, WHZ, and MUACZ) and malaria infection [15]. However, we noted that children with acute malnutrition (WAZ) seem to develop less the disease. This result is in line with observations made during some studies carried out in humans, such as the one conducted by Ndeba et al. (2008) in the DRC, which showed an inverse association between malnutrition and the prevalence of malaria infection [16]. However, the exact mechanism of this protective effect remains difficult to explain. According to O'brien et al. (2018), the potential mechanism of this relationship is twofold, namely, behavioural through the protection of some malnourished people by mothers and caregivers and biological through the immunomodulatory effect of nutritional status or the increase in the ability of malnourished children to produce certain cytokines in response to stimulation by malaria-specific antigens [15]. In this study, malnourished refugee children supplemented with multivitamins may their effect affects positively the immune system of these children. These vitamin supplements stimulate the immune system which leads to the protective effect observed in these children against malaria.

Infection with *Plamodium falciparum* tended to have significant influence on the level of anti-*Plasmodium falciparum* antibodies in children. This result is in line with results of Fillol et al. (2009) who found that the IgG level specific to *Plamodium falciparum* was positively correlated with the intensity of infection [8]. Indeed, the immune response against *Plasmodium falciparum* infection may not be closely related to children's diets. Our results showed that no anthropometric parameters (WAZ, HAZ, WHZ, WHZ, BAZ, and MUACZ) had a significant influence on the anti-Pf IgG *T* level in children. The association between nutritional status and the immune response to *Plasmodium falciparum* is highly controversial from one study to another [8, 17, 18]. Fillol et al. (2009) noted that only growth retardation (HAZ) influenced schizont-specific IgG levels and that weight loss had no effect on IgG levels [8]. However, no association had been found between specific antibodies and chronic or acute malnutrition in children in Tanzania [18]. The differences observed from one study to another may be due to the discrepancies observed in the designs of the different studies.

## 5. Conclusion

As a result of this work, it can be seen that malaria and malnutrition remain real public health problems in the Gado-badzéré refugee camp. Malaria and malnutrition rates were high in this population, and acute malnutrition was statistically less recurrent among uninfected children than among infected children. The infection tended to have significant influence on the level of anti-*Plasmodium falciparum* antibodies in children. Knowledge on the nutritional profile of the population would be of great benefit in setting up an appropriate health program. We therefore suggest that more standardized studies be conducted to highlight the effect of nutrition and micronutrients on immunological status.

## Figures and Tables

**Table 1:** Mean comparison of Specific immune response (anti-Pf IgG (*T*) according to nutritional parameters, infectious status, and serum iron of children.

Groups of children	n	Mean (IgG T)	Standard deviation	*P* value
WHZ > −2	22	0.97	0.45	0.051
WHZ < −2	20	0.74	0.30	
HAZ > −2	35	0.85	0.41	0.704
HAZ < −2	7	0.91	0.38	
WAZ > −2	31	0.89	0.41	0.35
WAZ < −2	13	0.77	0.34	
BAZ > −2	23	0.96	0.44	0.078
BAZ < −2	19	0.74	0.31	
MUACZ > −2	35	0.87	0.40	0.678
MUACZ < −2	9	0.81	0.39	
Pf positive	21	0.90	0.40	0.39
Pf negative	21	0.79	0.39	
Iron >0.5	26	0.83	0.39	0.85
Iron <0.5	5	0.79	0.54	

**Table 2:** Assessment of the risk of having IgG *T* anti-*Plasmodium falciparum* below the threshold value (0.4409) in children based on their nutritional status, infected status, and serum iron levels.

IgG *T* anti-Pf < 0,4409	n	Risk ratio	95% confidence interval		*P* Value
Groups of children			Lower	Upper	
WHZ > −2	22	1.259	0.245	6.473	0.783
WHZ < −2	20	0.794	0.154	4.082	
HAZ > −2	35	1.241	0.125	12.286	0.853
HAZ < −2	7	0.806	0.081	7.973	
WAZ > −2	31	1.058	0.177	6.303	0.951
WAZ < −2	13	0.945	0.159	5.634	
BAZ > −2	23	1.123	0.218	5.777	0.89
BAZ < −2	19	0.891	0.173	4.582	
MUACZ > −2	35	0.583	0.093	3.653	0.565
MUACZ < −2	9	1.714	0.274	10.736	
Pf positive	21	0.125	0.014	1.151	0.06
Pf negative	21	8	0.869	73.683	
Iron>0.5	26	0.273	0.034	2.188	0.221
Iron<0.5	5	3.667	0.457	29.419	

## Data Availability

The data supporting the findings of this study are available from the corresponding author Constant Anatole Pieme upon request.
